# Predicting Different Recurrence Risk in HNSCC Using Circulating Inflammatory and T‐Cell Biomarkers

**DOI:** 10.1002/lary.70334

**Published:** 2025-12-23

**Authors:** Riccardo Gili, Paola Lovino Camerino, Luca Lalli, Elisa Bellini, Chiara Tagliaferri, Filippo Bruno Lanza, Stefania Vecchio, Simone Caprioli, Giorgio Peretti, Lucia Del Mastro, Filippo Marchi

**Affiliations:** ^1^ Medical Oncology, Department of Internal Medicine and Medical Specialties University of Genova Genoa Italy; ^2^ Department of Medical Oncology UO Clinica di Oncologia Medica, IRCCS Ospedale Policlinico San Martino Genoa Italy; ^3^ Unit of Otorhinolaryngology‐Head and Neck Surgery IRCCS Ospedale Policlinico San Martino Genoa Italy; ^4^ Department of Surgical Sciences and Integrated Diagnostics (DISC) University of Genova Genoa Italy; ^5^ Unit of Translational Immunology IRCCS Foundation National Cancer Institute Milan Italy; ^6^ Radiology Unit IRCCS Ospedale Policlinico San Martino Genoa Italy

**Keywords:** biomarkers, head and neck squamous cell carcinoma, inflammatory, recurrence

## Abstract

**Objective:**

Head and neck squamous cell carcinoma (HNSCC) lacks reliable prognostic circulating biomarkers, and the role of the immune system in its progression remains incompletely understood. This study aimed to evaluate the prognostic value of systemic inflammatory and immune biomarkers in surgically treated patients with primary oral cavity (OCSCC) or laryngeal squamous cell carcinoma (LSCC).

**Methods:**

This retrospective single‐center study included 394 patients with OCSCC or LSCC who underwent surgery with curative intent. Preoperative blood samples were analyzed to assess platelet, neutrophil, lymphocyte, and monocyte counts, as well as T‐cell subpopulations (CD3+, CD4+, and CD8+). Composite ratios—including neutrophil‐to‐lymphocyte ratio (NLR), platelet‐to‐lymphocyte ratio (PLR), lymphocyte‐to‐monocyte ratio (LMR), and systemic immune‐inflammation index (SII)—were evaluated for their correlation with global (GR), local (LR), and distant (DR) recurrence risk.

**Results:**

Univariate analysis identified elevated platelet counts, PLR, SII, and neutrophil levels as significantly associated with increased GR (*p* = 0.0001, *p* = 0.0014, *p* = 0.0058 and *p* = 0.016, respectively) and LR risk, with the exception of neutrophil levels (*p* = 0.0002, *p* = 0.0008, *p* = 0.0072, respectively). In contrast, higher CD4+/CD3+ (*p* = 0.038) and CD4+/CD8+ (*p* = 0.045) ratios were associated with reduced DR risk. Multivariate analysis confirmed platelet count as an independent predictor for GR (*p* = 0.0001) and LR (*p* = 0.0002), while a high CD4+/CD8+ ratio (*p* = 0.045) was independently protective against DR.

**Conclusion:**

These findings highlight the prognostic relevance of platelet‐related inflammatory markers and circulating T‐cell ratios in recurrence risk among HNSCC patients. The differential associations with local versus distant recurrence underscore the complex interplay between systemic inflammation and adaptive immunity. Prospective validation is warranted to confirm these biomarkers' utility.

**Level of Evidence:**

4.

## 1 Introduction

In head and neck squamous cell carcinoma (HNSCC), apart from human papillomavirus (HPV) and Epstein–Barr virus (EBV), there is still a lack of circulating biomarkers with established prognostic value or routine clinical application. Furthermore, the role of the immune system in the etiopathogenesis and progression of HNSCC remains incompletely understood.

It is well established that systemic inflammation is closely linked to tumorigenesis and cancer progression. For instance, macrophages, neutrophils, and T‐helper 2 (Th2) lymphocytes release cytokines that promote chronic inflammation, whereas CD8+ lymphocytes, natural killer cells, and other cytotoxic effectors exert antitumor activity [1, 2].

Recently, interest in systemic inflammatory markers as prognostic indicators in various solid tumors has increased. Multiple studies have demonstrated an association between poor disease control and survival and elevated neutrophil‐to‐lymphocyte ratio (NLR), which reflects the dynamic balance between innate (neutrophils) and adaptive (lymphocytes) immune responses [3]. Other markers, including platelet‐to‐lymphocyte ratio (PLR), systemic immune‐inflammation index (SII), and lymphocyte‐to‐monocyte ratio (LMR), have also shown correlations with survival outcomes, albeit with less consistent evidence [4, 5, 6, 7].

Moreover, the interplay between cancer and peripheral T‐cell subpopulations results in varying circulating levels of CD3+, CD4+, and CD8+ lymphocytes depending on disease stage (early, advanced, or metastatic) [8].

Despite these findings, the prognostic significance of immune response markers in HNSCC remains controversial, with limited consensus regarding specific circulating inflammatory biomarkers.

Our study aims to investigate the role of the immune system in HNSCC patients by assessing the correlation between biomarker levels and risks of global recurrence (GR), local recurrence (LR), and distant recurrence (DR).

## 2 Materials and Methods

This retrospective, single‐center study was conducted by the Medical Oncology 2 Unit and the Otolaryngology–Head and Neck Surgery Unit at IRCCS Ospedale Policlinico San Martino, University of Genova, in Genova, Italy. Ethical review and approval were not required in accordance with the national and institutional policy; however, every patient preoperatively signed informed consent in accordance with the Declaration of Helsinki. We enrolled 805 patients treated with curative intent for primary HNSCC—oral cavity (OCSCC) or larynx (LSCC)—between 2015 and 2022. This study had the following objectives: (1) to evaluate the correlation between biomarker values and GR, DR, and LR risk, (2) to evaluate any statistically significant difference in biomarker values in terms of GR, DR, and LR by stratifying it on subsites, and (3) on tumor stage.

LR was defined as the reappearance of tumor disease at or near the site of the primary tumor (local recurrence) or within the regional lymphatic drainage areas (regional recurrence) after a period of complete remission following initial treatment.

Distant recurrence was defined as the reappearance of tumor disease at anatomical sites that are not contiguous with the primary tumor region or its regional lymphatic drainage.

To achieve these, the inclusion criteria were: (1) primary OCSCC or larynx squamous cell carcinoma (LSCC), tumor stage classified according to the 8th Edition of the AJCC UICC TNM staging system [9] (pT1‐pT4/pN1‐N3); (2) curative surgical treatment with or without adjuvant treatment; (3) pre‐operative blood samples data availability; (4) minimum follow‐up of 1 year. Exclusion criteria were: (1) distant metastasis; (2) previous cancer other than HNSCC; (3) immunological disorders; (4) long‐term steroid treatments history; (5) chronic inflammatory conditions history; (6) bacterial and/or viral infections at the time of blood sample collection. Three hundred and ninety‐four patients met the inclusion criteria. Preoperative workup, treatment, and follow‐up. All patients underwent routine full blood sampling: platelets (PLT), white blood cells (WBC), neutrophils (NEU), lymphocytes (LYM), and monocyte (MON) counts, quantitative analysis of CD3+, CD4+, and CD8+ T lymphocyte subpopulations (performed by cytofluorometry—FACS CANTO II Cytometer, Becton Dickinson, San Jose, CA, USA).

In our institute's clinical practice, in addition to blood counts and all other tests deemed necessary in the preoperative pathway for patients who are candidates for radical surgery, the CD3+, CD4+, and CD8+ T lymphocyte subpopulations assessment is usually performed.

The NLR was defined as the absolute neutrophil divided by absolute lymphocyte counts. The PLR was considered as the absolute platelet divided by absolute lymphocyte counts. The LMR was considered as the absolute lymphocyte divided by absolute monocyte counts. The systemic immune‐inflammation index (SII) was defined as neutrophils×platelets/lymphocytes. We also evaluated the CD4+/CD3+, CD8+/CD3+, and CD4+/CD8+ ratios defined as the ratios of the absolute counts of the respective T‐cell subpopulations. All patients underwent complete surgical excision of the primary tumor +/− neck dissection and adjuvant therapy according to disease clinical stage, following NCCN guidelines and the patient's performance status [10].

The study was approved by “Comitato Etico Liguria”, our Institutional Review Board.

## 3 Statistical Analysis

The biomarkers' impact on tumor stage was assessed using the Mann–Whitney/Wilcoxon test. Cox univariate models were employed to evaluate associations with biomarkers and three outcomes: GR, LR, and DR. Survival analyses were conducted using Kaplan–Meier curves, stratified by tumor stage and location. Multivariate Cox models with stepwise selection were used to identify the most significant predictors for the three outcomes. A two‐sided significance level of 5% was adopted for all analyses. Statistical computations were performed using R software (version 4.4.1, R Foundation for Statistical Computing, Vienna, Austria). In the models used, each biomarker was considered as a continuous variable, making it unnecessary to identify specific cutoffs.

## 4 Results

**FIGURE 1:**
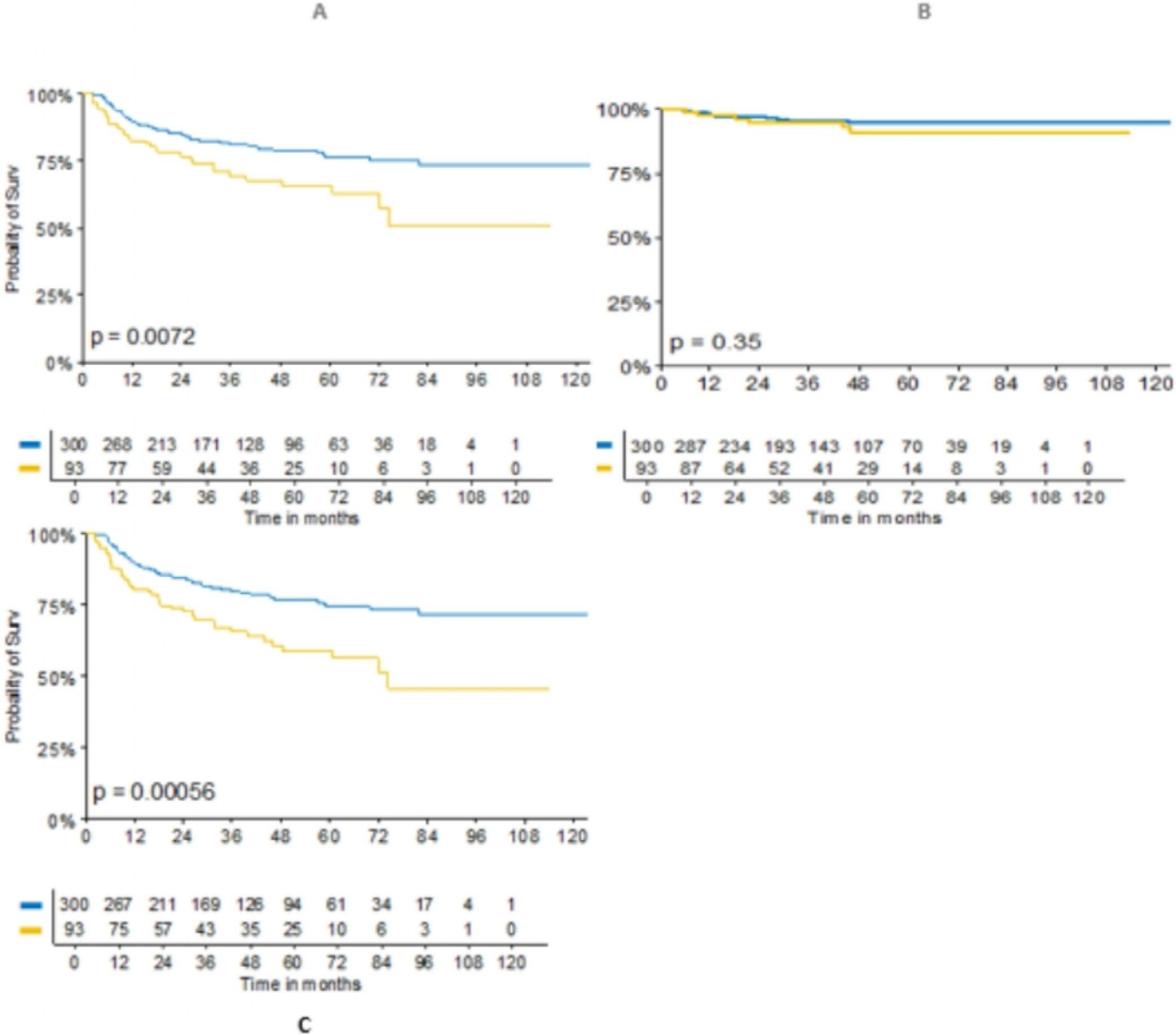
(a) Local. (b) Distant. (c) Global recurrence risk comparing laryngeal (blue) and oral (yellow) squamous cell carcinoma. [Color figure can be viewed in the online issue, which is available at www.laryngoscope.com]

Of the 394 patients who met inclusion criteria, the median follow‐up (mFUP) period was 47.77 (26.84–71.10) months. The primary site was LSCC for 300 patients and OCSCC for 94 patients. Patients' characteristics are summarized in Tables 1 and 2. We analyzed the risk of recurrence for each individual variable, either by considering GR, LR and DR. Next, specific analyses were done clustering patients according to different subsite (larynx and oral cavity) and stage (Stages 1–2 and Stage 3–4), to assess the role of biomarkers in patients with similar characteristics. Finally, a multivariate analysis was performed for risk of recurrence, without stratifying patients by stage or subsite. Global, distant and local recurrence risk Univariate analysis showed that high platelet count (HR = 1.14, *p* = 0.0001), elevated SII (HR = 1.12, *p* = 0.0014), high PLR (HR = 1.17, *p* = 0.0058), and increased neutrophil levels (HR = 1.30, *p* = 0.016) were linked with greater GR risk. Additionally, higher CD4+/CD3+ (HR = 0.41, *p* = 0.038) and CD4+/CD8+ (HR = 0.25, *p* = 0.045) ratios correlated with a decreased risk of DR, while high platelet count (HR = 1.14, *p* = 0.0002), elevated SII (HR = 1.12, *p* = 0.0008) and high PLR (HR = 1.17, *p* = 0.0072) were also linked with greater LR risk (Table 3). Multivariate analysis confirmed the prognostic role of platelet, both for LR (HR = 1.14, *p* = 0.0002) and GR (HR = 1.14, *p* = 0.0001) risk, and the protective role of a high CD4+/CD8+ (HR = 0.25, *p* = 0.045) ratio considering the DR risk. Larynx and Oral Cavity It was observed that OCSCC patients had a higher recurrence rate considering both GR (HR 2.00, 95% CI 1.33–2.99, *p* = 0.0007) and LR (HR 1.79, 95% CI 1.16–2.76, *p* = 0.008), while no statistically significant differences were observed for DR (Figure 1). The analysis of biomarkers in LSCC showed that high platelet count (HR = 1.13, 95% CI 1.05–1.22, *p* = 0.0012), elevated SII (HR = 1.13, 95% CI 1.04–1.22, *p* = 0.0027), high PLR (HR = 1.15, 95% CI 1.02–1.29, *p* = 0.0168) and increased neutrophil levels (HR 1.34, 95% CI 1.01–1.79, *p* = 0.0388) were linked with greater LR risk (Table 4). In LSCC, elevated platelet count (HR = 1.13, 95% CI 1.05–1.22, *p* = 0.0012), SII (HR = 1.13, 95% CI 1.04–1.22, p = 0.0027), PLR (HR = 1.15, 95% CI 1.02–1.29, *p* = 0.0168), and neutrophil levels (HR = 1.34, 95% CI 1.01–1.79, p = 0.0388) were associated with an increased risk of LR (Table 4). Considering only the DR risk, no biomarkers were shown to be statistically significant, while high platelet count (HR = 1.13, 95% CI 1.04–1.22, *p* = 0.0023), elevated SII (HR = 1.13, 95% CI, *p* = 0.0030) and high PLR (HR = 1.16, 95% CI 1.03–1.30, *p* = 0.0128) were linked with greater GR risk (Table 4). For OCSCCs only CD4+ were correlated with a decreased risk of DR, while no biomarkers proved statistically significant in predicting the risk of recurrence for either LR or GR (Table 4). Early‐stage versus Advanced‐stage Patients were divided into two different groups: low stage (Stages I and II) and high stage (Stages III and IV) SCC, regardless of subsite. As expected, the LR and DR were higher in advanced LSCC/OCSCC. Regarding high stage SCC, our analysis showed that high platelet count (HR = 1.14, 95% CI 1.03–1.27, *p* = 0.0081), elevated SII (HR = 1.12, 95% CI 1.00–1.25, *p* = 0.0456) and high PLR (HR = 1.20, 95% CI 1.04–1.39, *p* = 0.0123) were associated with greater LR risk, but also that high platelet count (HR = 1.14, 95% CI 1.04–1.26, *p* = 0.0056) and high PLR (HR = 1.18, 95% CI 1.02–1.37, *p* = 0.0230) were linked with greater GR risk, while no biomarkers was statistically significant in predicting the risk of DR, or any type of recurrence in low stage patients (Table 5).

**TABLE 1:** Patients' characteristics according to the subsite.

Larynx (n° 300)			Oral cavity (n° 94)		
N/A	n°	2	N/A	n°	0
pT1	n°	133	pT1	n°	28
pT2	n°	53	pT2	n°	37
pT3	n°	69	pT3	n°	14
pT4	n°	43	pT4	n°	5
c/pN0	n°	264	c/pN0	n°	68
pN1	n°	14	pN1	n°	12
pN2	n°	13	pN2	n°	10
pN3	n°	7	pN3	n°	4
Stage I	n°	133	Stage I	n°	24
Stage II	n°	49	Stage II	n°	28
Stage III	n°	68	Stage III	n°	21
Stage IV	n°	48	Stage IV	n°	21
Stage IVa	n°	41	Stage IVa	n°	16
Stage IVb	n°	7	Stage IVb	n°	5
Relapse	n°	67	Relapse	n°	37
LR	n°	54	LR	n°	31
DR	n°	5	DR	n°	6
LR + DR	n°	8	LR + DR	n°	0

**TABLE 2:** Different patterns of recurrence according to the stage.

Low stage (n° 181)			High stage (n° 117)		
Relapse	n°	30	Relapse	n°	36
LR	n°	29	LR	n°	24
DR	n°	0	DR	n°	6
LR + DR	n°	1	LR + DR	n°	6

**TABLE 3:** Correlation between inflammatory biomarkers and global, distant recurrence risk.

Var	HR	95% CI	*p*
Global recurrence			
PLT	1.1416	1.0674–1.2209	0.0001*
SII	1.1220	1.0490–1.2000	0.0008*
PLR	1.1734	1.0473–1.3146	0.0058*
NEU	1.3052	1.0490–1.6238	0.0169*
WBC	1.2231	0.9657–1.5491	0.0948
NLR	1.1001	0.9360–1.2930	0.2469
CD4_CD3	0.9283	0.7112–1.2118	0.5844
MON	0.9806	0.9120–1.0543	0.5961
CD3	1.0752	0.8083–1.4301	0.6186
CD4_CD8	0.9539	0.7387–1.2319	0.7176
CD4	1.0415	0.8005–1.3551	0.7621
LMR	0.9748	0.7709–1.2327	0.8313
LYM	0.9721	0.7451–1.2683	0.8349
CD8_CD3	0.9890	0.8747–1.1181	0.8595
CD8	0.9904	0.8532–1.1495	0.8986
Distant recurrence			
CD4_CD3	0.4151	0.1808–0.9529	0.0381*
CD4_CD8	0.2501	0.0645–0.9696	0.0450*
NEU	1.4547	0.9403–2.2507	0.0923
CD4	0.5047	0.1902–1.3393	0.1697
NLR	1.1669	0.8531–1.5961	0.3342
WBC	1.2634	0.7627–2.0927	0.3639
PLR	1.0802	0.8235–1.4169	0.5775
CD3	0.7743	0.3030–1.9789	0.5932
CD8_CD3	1.0405	0.8978–1.2058	0.5981
SII	1.0477	0.8662–1.2672	0.6314
CD8	1.0329	0.7881–1.3538	0.8145
MON	0.9875	0.8860–1.1005	0.8195
PLT	1.0276	0.8016–1.3172	0.8300
LYM	0.9707	0.5441–1.7320	0.9199
LMR	0.9872	0.5923–1.6453	0.9606
Local recurrence			
PLT	1.1437	1.0669–1.2259	0.0002*
SII	1.1224	1.0459–1.2045	0.0014*
PLR	1.1768	1.0451–1.3250	0.0072*
NEU	1.2401	0.9757–1.5763	0.0786
WBC	1.1752	0.9100–1.5177	0.2159
NLR	1.0854	0.9082–1.2971	0.3676
CD3	1.1173	0.8347–1.4955	0.4561
CD4	1.0897	0.8341–1.4235	0.5288
MON	0.9818	0.9127–1.0562	0.6228
LMR	0.9539	0.7416–1.2268	0.7129
CD8_CD3	0.9778	0.8412–1.1365	0.7696
CD4_CD3	0.9706	0.7398–1.2733	0.8294
LYM	0.9814	0.7397–1.3021	0.8967
CD8	0.9924	0.8539–1.1535	0.9212
CD4_CD8	0.9933	0.7681–1.2844	0.9591

*Note*: * represents *p* < 0.05.

**TABLE 4:** Correlation between inflammatory biomarkers and global, distant, and local recurrence risk considering the subsite.

Larynx					Oral cavity	
Var	HR	95% CI	*p*	HR	95% CI	*p*
Global recurrence						
PLT	1.1340	1.0510–1.2236	0.0012	1.1393	0.9501–1.3662	0.1592
WBC	1.2141	0.8999–1.6380	0.2042	1.3761	0.9493–1.9946	0.0919
NEU	1.3494	1.0156–1.7929	0.0388	1.3373	0.9593–1.8641	0.0864
LYM	0.9619	0.6790–1.3625	0.8267	1.0954	0.7232–1.6591	0.6671
MON	0.9914	0.8918–1.1020	0.8723	0.9745	0.8356–1.1366	0.7424
NLR	1.1047	0.9052–1.3483	0.3271	1.1611	0.8697–1.5502	0.3111
PLR	1.1524	1.0259–1.2944	0.0168	1.1727	0.8356–1.6458	0.3568
SII	1.1328	1.0443–1.2288	0.0027	1.2475	0.9749–1.5964	0.0788
LMR	0.8444	0.5992–1.1900	0.3340	1.0923	0.7585–1.5731	0.6351
CD4_CD3	1.0743	0.7426–1.5543	0.7037	0.7196	0.4543–1.1397	0.1607
CD8_CD3	1.0120	0.7018–1.4593	0.9490	0.9397	0.7320–1.2063	0.6256
CD4_CD8	1.0733	0.7629–1.5101	0.6847	0.8225	0.5457–1.2396	0.3504
CD3	1.0733	0.7666–1.5028	0.6802	1.4214	0.8040–2.5131	0.2265
CD4	1.1117	0.8152–1.5159	0.5035	1.0259	0.6357–1.6557	0.9166
CD8	1.0059	0.7094–1.4263	0.9738	0.9711	0.8055–1.1708	0.7588
Distant recurrence						
PLT	1.0867	0.9402–1.2560	0.2605	0.8669	0.3930–1.9122	0.7234
WBC	1.3665	0.7551–2.4732	0.3022	1.1347	0.4371–2.9458	0.7951
NEU	1.4803	0.8628–2.5397	0.1544	1.4768	0.7152–3.0493	0.2919
LYM	1.2902	0.6646–2.5045	0.4515	0.4023	0.1039–1.5578	0.1874
MON	1.0195	0.8724–1.1914	0.8080	0.3980	0.0837–1.8931	0.2469
NLR	1.0853	0.7139–1.6497	0.7018	1.5743	0.9215–2.6894	0.0967
PLR	1.0479	0.7866–1.3961	0.7491	1.2631	0.5612–2.8428	0.5726
SII	1.0314	0.8036–1.3237	0.8084	1.2784	0.7214–2.2654	0.4001
LMR	0.9560	0.4818–1.8969	0.8976	0.9908	0.4045–2.4269	0.9839
CD4_CD3	0.5112	0.1811–1.4430	0.2051	0.2707	0.0507–1.4441	0.1261
CD8_CD3	1.9347	0.8561–4.3725	0.1127	1.0158	0.7423–1.3899	0.9222
CD4_CD8	0.2891	0.0505–1.6553	0.1634	0.2155	0.0197–2.3524	0.2082
CD3	1.3024	0.4510–3.7611	0.6253	0.2633	0.0317–2.1902	0.2170
CD4	0.9229	0.3106–2.7423	0.8851	0.1191	0.0150–0.9432	0.0439
CD8	1.6220	0.7485–3.5150	0.2203	0.8788	0.2483–3.1106	0.8413
Local recurrence						
PLT	1.1329	1.0456–1.2274	0.0023	1.1649	0.9670–1.4032	0.1081
WBC	1.1218	0.8120–1.5498	0.4857	1.4059	0.9405–2.1017	0.0967
NEU	1.2716	0.9345–1.7302	0.1263	1.2846	0.8834–1.8681	0.1899
LYM	0.8907	0.6143–1.2916	0.5417	1.2704	0.8257–1.9546	0.2762
MON	0.9867	0.8748–1.1130	0.8278	0.9823	0.9039–1.0675	0.6743
NLR	1.1152	0.9066–1.3718	0.3019	1.0522	0.7402–1.4959	0.7766
PLR	1.1607	1.0321–1.3052	0.0128	1.1371	0.7828–1.6519	0.5000
SII	1.1351	1.0438–1.2344	0.0030	1.2219	0.9282–1.6083	0.1530
LMR	0.8121	0.5651–1.1672	0.2608	1.1192	0.7510–1.6680	0.5802
CD4_CD3	1.0582	0.7296–1.5348	0.7656	0.8207	0.5180–1.3003	0.4001
CD8_CD3	1.0319	0.7149–1.4894	0.8669	0.9040	0.6225–1.3127	0.5959
CD4_CD8	1.0632	0.7521–1.5031	0.7285	0.9075	0.6049–1.3614	0.6389
CD3	1.0488	0.7452–1.4761	0.7848	1.7276	0.9493–3.1439	0.0735
CD4	1.0828	0.7897–1.4849	0.6213	1.2653	0.7799–2.0528	0.3405
CD8	1.0029	0.7043–1.4282	0.9871	0.9754	0.8071–1.1789	0.7969

**TABLE 5:** Correlation between inflammatory biomarkers and global, distant, and local recurrence risk considering the stage.

Var	Stage 1–2			Stage 3–4		
	HR	95% CI	*p*	HR	95% CI	*p*
Global recurrence						
PLT	0.8921	0.5429–1.4661	0.6524	1.1484	1.0413–1.2665	0.0056
WBC	1.2611	0.8105–1.9622	0.3037	1.0019	0.6973–1.4395	0.9919
NEU	1.4391	0.9282–2.2314	0.1038	1.1005	0.7713–1.5703	0.5975
LYM	0.9063	0.5416–1.5165	0.7080	0.9568	0.6150–1.4888	0.8449
MON	1.2023	0.7569–1.9100	0.4352	0.9642	0.8430–1.1029	0.5949
NLR	1.3044	0.9047–1.8807	0.1546	0.9571	0.7211–1.2703	0.7616
PLR	0.8901	0.5796–1.3668	0.5946	1.1858	1.0238–1.3736	0.0230
SII	1.1504	0.7640–1.7322	0.5023	1.1150	0.9961–1.2480	0.0585
LMR	0.8284	0.4601–1.4916	0.5304	0.9279	0.6363–1.3529	0.6972
CD4_CD3	1.0786	0.6586–1.7665	0.7636	1.1024	0.6092–1.9949	0.7473
CD8_CD3	0.9862	0.5923–1.6423	0.9575	1.0017	0.5922–1.6945	0.9949
CD4_CD8	0.8745	0.5387–1.4196	0.5874	1.3541	0.8051–2.2772	0.2531
CD3	1.0194	0.6405–1.6225	0.9355	1.1787	0.7185–1.9338	0.5150
CD4	1.1054	0.7160–1.7065	0.6510	1.1438	0.7136–1.8335	0.5767
CD8	0.9515	0.5923–1.5285	0.8371	1.1489	0.7059–1.8698	0.5765
Distant recurrence						
PLT	1.2862	0.1469–11.2628	0.8201	1.0156	0.7599–1.3572	0.9168
WBC	2.7107	0.4651–15.7981	0.2675	0.9835	0.5483–1.7643	0.9556
NEU	2.8262	0.5093–15.6837	0.2347	1.0965	0.6206–1.9374	0.7511
LYM	0.5128	0.0352–7.4818	0.6253	1.3354	0.6662–2.6766	0.4150
MON	15.9831	0.222–1149.770	0.2039	0.9612	0.750–1.231	0.7542
NLR	1.7671	0.5430–5.7503	0.3443	0.8822	0.5203–1.4958	0.6417
PLR	1.2480	0.3620–4.3021	0.7257	0.9649	0.6290–1.4803	0.8701
SII	1.8168	0.6323–5.2205	0.2676	0.8917	0.5285–1.5044	0.6675
LMR	< 0.0001	0.0001–8.9267	0.1125	1.2881	0.7438–2.2305	0.3662
CD4_CD3	0.6676	0.0567–7.8561	0.7481	0.5072	0.1531–1.6807	0.2668
CD8_CD3	14.0437	0.382–516.348	0.1508	1.4249	0.5222–3.8880	0.4893
CD4_CD8	0.0583	0.0002–15.7636	0.3198	0.3691	0.0578–2.3590	0.2923
CD3	0.1702	0.0049–5.8653	0.3269	1.9182	0.5834–6.3077	0.2835
CD4	0.1329	0.0025–7.1501	0.3210	1.2142	0.3770–3.9108	0.7450
CD8	0.9880	0.0862–11.3234	0.9923	2.6362	0.8745–7.9469	0.0851
Local recurrence						
PLT	0.8921	0.5429–1.4661	0.6524	1.1496	1.0369–1.2744	0.0081
WBC	1.2611	0.8105–1.9622	0.3037	0.8932	0.5905–1.3511	0.5927
NEU	1.4391	0.9282–2.2314	0.1038	1.0058	0.6687–1.5128	0.9779
LYM	0.9063	0.5416–1.5165	0.7080	0.8472	0.5171–1.3880	0.5104
MON	1.2023	0.7569–1.9100	0.4352	0.9627	0.8251–1.1233	0.6292
NLR	1.3044	0.9047–1.8807	0.1546	0.9820	0.7262–1.3278	0.9058
PLR	0.8901	0.5796–1.3668	0.5946	1.2062	1.0415–1.3969	0.0123
SII	1.1504	0.7640–1.7322	0.5023	1.1237	1.0023–1.2598	0.0456
LMR	0.8284	0.4601–1.4916	0.5304	0.8526	0.5585–1.3015	0.4599
CD4_CD3	1.0786	0.6586–1.7665	0.7636	1.0644	0.5829–1.9434	0.8391
CD8_CD3	0.9862	0.5923–1.6423	0.9575	1.0398	0.6141–1.7606	0.8846
CD4_CD8	0.8745	0.5387–1.4196	0.5874	1.3410	0.7874–2.2838	0.2801
CD3	1.0194	0.6405–1.6225	0.9355	1.1268	0.6768–1.8759	0.6462
CD4	1.1054	0.7160–1.7065	0.6510	1.0876	0.6676–1.7719	0.7359
CD8	0.9515	0.5923–1.5285	0.8371	1.1406	0.6930–1.8773	0.6049

## 5 Discussion

Chronic inflammation is a well‐established hallmark of HNSCC [11, 12], driving sustained tissue damage and significantly contributing to tumor initiation and progression [13, 14]. The causes of chronic inflammation are multifactorial, including alcohol consumption, smoking history, and resultant hypoxia mediated through activation of hypoxia‐inducible factor‐1 (HIF‐1) and nuclear factor κB (NF‐κB), as well as the tumor microenvironment itself, which produces inflammatory cytokines and chemokines [8, 15, 16]. Notably, interleukins such as IL‐4, IL‐5, and IL‐1α (which promotes proliferation of cancer‐associated fibroblasts), tumor necrosis factor‐alpha (TNF‐α), NF‐κB, transforming growth factor‐beta (TGF‐β), and activation of the JAK/STAT3 pathway have been implicated. These factors also inhibit apoptosis through blockade of the FAS‐associated death domain protein [11].

Tumors are infiltrated by leukocytes, particularly T‐cells, whose role remains complex due to the functional heterogeneity of tumor‐infiltrating lymphocytes (TILs) [17, 18]. Despite their presence, these cells seem to have limited capacity to initiate systemic inflammatory responses, and circulating monocytes often exhibit impaired responsiveness to cytokine stimulation [8].

To better understand the interactions among head and neck tumors, the immune system, and inflammation, several studies have assessed the prognostic value of circulating inflammatory biomarkers, with PLR and NLR being the most extensively studied. However, findings have been inconsistent and marked by methodological heterogeneity. To reduce potential selection bias, our study excluded patients undergoing immunomodulatory therapy or those affected by autoimmune diseases, second primary malignancies, or chronic inflammatory conditions.

For the first time, we evaluated the correlation between circulating inflammatory biomarkers and recurrence risk—including GR, LR, and DR—not only in the entire cohort but also stratified by tumor subsite (larynx and oral cavity) and disease stage (low vs. high). Our findings show that platelets and platelet‐related indices, such as PLR and SII, significantly correlate with GR and LR risk in univariate analyses. These associations were confirmed within the LSCC subgroup (Table 4) and among patients with advanced‐stage disease (Table 5). Multivariate analysis further validated the prognostic role of platelets in predicting GR and LR across the entire study population.

The oncogenic role of platelets is well documented: they interact with tumor cells to promote survival and hematogenous metastasis by shielding tumor cells from natural killer (NK) cell‐mediated lysis, facilitating metastatic niche formation, and acting as chemoattractants [4, 5]. Additionally, platelets induce epithelial‐mesenchymal transition and enhance tumor angiogenesis through secretion of TGF‐β and vascular endothelial growth factor (VEGF), respectively [4]. The prognostic relevance of PLR has been reported by Takenaka et al. [4], Bardash et al. [19], Zhou et al. [20], Chen et al. [21], and Wang et al. concerning SII [6].

Our study is the first to examine distinct recurrence patterns, recognizing that local and distant recurrences arise from fundamentally different mechanisms, which may be reflected in differential expression of circulating inflammatory biomarkers.

Contrary to Acharya et al. [22], who found a relationship between PLR and survival in OCSCC, our study did not observe a significant correlation in this subgroup, potentially due to limited sample size.

Neutrophils play a central role in carcinogenesis, notably through production of granulocyte‐macrophage colony‐stimulating factor (GM‐CSF), which promotes neutrophil proliferation and differentiation [23]. While neutrophils contribute to tumor control by releasing cytotoxic granules and recruiting other antitumor immune effectors, tumor‐derived factors can polarize neutrophils towards a pro‐tumorigenic phenotype, facilitating tumor progression and metastasis. Consequently, elevated neutrophil counts in blood and tumor tissue have been associated with worse outcomes in cancer patients [23, 24].

NLR reflects the dynamic interplay between innate and adaptive immunity. Elevated NLR correlates with various inflammatory conditions and is associated with metastatic potential, tumor stage, and lymphatic invasion [3]. Several meta‐analyses have evaluated the prognostic significance of pretreatment NLR in HNSCC across different subsites and metastatic statuses [25, 26, 27, 28]. In our cohort, only elevated neutrophil counts were associated with increased GR risk, while NLR did not correlate with recurrence risk in any subgroup. This supports the notion that preoperative PLR may serve as a superior prognostic marker compared to NLR, consistent with Chen et al. [29].

The lack of association between NLR and prognosis may be due to: (1) variability in cutoff values and absence of standardized thresholds, (2) our analysis treating biomarkers as continuous variables without predefined cutoffs, (3) retrospective study design, (4) unclear patient selection criteria in existing literature, and (5) predominance of Asian cohorts with distinct patient characteristics in prior studies.

Regarding peripheral T‐cell subsets, data on their prognostic role in HNSCC are limited. Increased CD4+ counts alongside decreased CD3+ and CD8+ populations have been linked to favorable outcomes [8, 30], a trend also observed in tumor‐infiltrating lymphocytes [18, 31, 32, 33]. Our study identified higher CD4+/CD3+, and CD4+/CD8+ ratios as significantly associated with reduced DR risk in both univariate and multivariate analyses, corroborating prior reports by Marchi et al. and Drennan et al. [8, 28]. This is the first investigation assessing the relationship between T‐cell ratios and distant recurrence risk, potentially reflecting systemic immunosurveillance mechanisms inhibiting metastatic spread.

Limitations of our study include its single‐center retrospective design and lack of OS data, which is a standard prognostic endpoint. Prospective studies are warranted to further clarify the prognostic utility of inflammatory biomarkers in terms of DFS and OS. Currently, an ongoing Italian multicenter prospective trial (NCT06809673) is evaluating the correlation between prognosis and various inflammatory biomarkers in HNSCC patients undergoing surgery with or without adjuvant therapy. Evidence from a prospective study of circulating markers related to survival outcomes, in a setting where available markers are lacking, could better stratify patients and define personalized treatments, especially in the case of elderly patients with comorbidities, or to propose de‐escalation/escalation strategies.

In addition, we have deliberately chosen to analyze biomarkers as continuous variables and not to apply specific cut‐off values to minimize information loss and avoid arbitrary dichotomization, which is widely recommended for etiological and prognostic modeling. The identification of optimal thresholds and their validation in an independent cohort require a sufficiently large, separate dataset, which was beyond the scope of this retrospective single‐center analysis. Nevertheless, this remains a planned objective of our ongoing multicenter prospective study (NCT06809673), where the most relevant biomarkers identified here will be re‐evaluated for optimal cut‐offs and externally validated.

## 6 Conclusion

This study highlights the prognostic value of systemic inflammatory biomarkers—especially platelet count, PLR, and SII—in predicting recurrence in HNSCC, particularly in laryngeal and advanced‐stage tumors. Additionally, higher CD4+/CD8+ and CD4+/CD3+ ratios were associated with lower distant recurrence risk, suggesting a role for adaptive immunity in limiting metastasis. By distinguishing between local and distant recurrence, this work offers a more detailed understanding of biomarker significance and supports their use in preoperative risk assessment. Further prospective studies are needed to validate these findings and explore their clinical application.

## Funding

The authors have nothing to report.

## Conflicts of Interest

The authors declare no conflicts of interest.

## Data Availability

The data that support the findings of this study are available from the corresponding author upon reasonable request.
